# Automatic segmentation of chest X-ray images via deep-improved various U-Net techniques

**DOI:** 10.1177/20552076251366855

**Published:** 2025-08-06

**Authors:** Sedat Orenc, Mehmet Sirac Ozerdem, Emrullah Acar, Musa Yilmaz

**Affiliations:** 1 Electrical-Electronics Engineering Department, 187432 Batman University, Batman, Turkey; 2 Electrical-Electronics Engineering Department, 37507 Dicle University, Diyarbakır, Turkey; 3 Center for Environmental Research and Technology, 8790 University of California, Bourns College of Engineering, Riverside, CA, USA

**Keywords:** Image processing, medical image segmentation, U-Net, chest X-ray, deep learning, dice coefficient, intersection over union

## Abstract

**Objectives:**

Accurate segmentation of medical images is vital for effective disease diagnosis and treatment planning. This is especially important in resource-constrained environments. This study aimed to evaluate the performance of various U-Net-based deep learning architectures for chest X-ray (CXR) segmentation and identify the most effective model in terms of both accuracy and computational efficiency.

**Methods:**

We assessed the segmentation performance of eight U-Net variants: U-Net7, U-Net9, U-Net11, U-Net13, U-Net16, U-Net32, U-Net64, and U-Net128. The evaluation was conducted using a publicly available CXR dataset categorized into normal, COVID-19, and viral pneumonia classes. Each image was paired with a corresponding segmentation mask. Image preprocessing involved resizing, noise filtering, and normalization to standardize input quality. All models were trained under identical experimental conditions to ensure a fair comparison. Performance was evaluated using two key metrics: Intersection over Union (IoU) and Dice Coefficient (DC). Additionally, computational efficiency was measured by comparing the total number of trainable parameters and the training time for each model.

**Results:**

U-Net9 achieved the highest performance among all tested models. It recorded a DC of 0.98 and an IoU of 0.96, outperforming both shallower and deeper U-Net architectures. Models with increased depth or filter width, such as U-Net128, showed diminishing returns in accuracy. These models also incurred significantly higher computational costs. In contrast, U-Net16 and U-Net32 demonstrated reduced segmentation accuracy compared to U-Net9. Overall, U-Net9 provided the optimal balance between precision and computational efficiency for CXR segmentation tasks.

**Conclusion:**

The U-Net9 architecture offers a superior solution for CXR image segmentation. It combines high segmentation accuracy with computational practicality, making it suitable for real-world applications. Its implementation can support radiologists by enabling faster and more reliable diagnoses. This can lead to improved clinical decision-making and reduced diagnostic delays. Future work will focus on integrating U-Net9 with multimodal imaging data, such as combining CXR with computerized tomography or MRI scans. Additionally, exploration of advanced architectures, including attention mechanisms and hybrid models, is planned to further enhance segmentation performance.

## Introduction

Recently, Artificial intelligence (AI) has progressively integrated into human life. It is designed to imitate the human cognitive process.^1^ Among the various branches of AI, deep learning has emerged as a leading and influential approach. It is defined by using neural networks with multiple layers. These networks enable advanced pattern recognition and support complex decision-making processes. It has attracted significant attention due to its exceptional ability to autonomously learn hierarchical representations from complex data. It leads to breakthroughs in various domains, including face recognition, speech recognition, object detection, and image segmentation.^2^

Image segmentation is a vital application of deep learning and plays an essential role in computer vision. Deep learning models, particularly convolutional neural networks (CNNs), have consistently demonstrated superior effectiveness in performing accurate and reliable segmentation.^3^ Image segmentation has become an extensively researched area. It is mainly used in visual applications and computer vision tasks. It is a fundamental process in computer vision. Image segmentation involves classifying each pixel within an image into specific categories. This approach represents a method of pixel-level classification.^4^ The primary objective underlying image segmentation is the partitioning of an image into multiple coherent and analyzable segments characterized by similar or identical features. In other words, the fundamental target of image segmentation is to simplify the representation of an image or make it more meaningful for analysis.^5^

Within the domain of segmentation methods, two prominent categories exist: Semantic segmentation and instance segmentation methods.^6^ Semantic segmentation involves classifying each pixel in an image into meaningful predefined classes. The main objective of semantic segmentation is to understand the overall context of an image by assigning a specific label to every pixel. This process facilitates a comprehensive analysis of the scene. It enables computers to recognize and differentiate various objects or regions. Semantic segmentation is widely applied across various fields, including autonomous vehicles, medical imaging, and satellite image analysis.^7^ On the contrary, instance segmentation surpasses semantic segmentation by not only categorizing pixels into classes but also distinguishing between individual instances or occurrences of objects within the same class. The main objective of instance segmentation is to provide a detailed understanding of each specific object in an image. This process enables a comprehensive analysis of the scene. It allows computers to recognize and distinguish between various objects or regions.^8^ Both methods play essential roles in various computer vision applications. They significantly contribute to advancements in fields such as medical diagnostics and image segmentation.^9^

One of the most widely used image segmentation methods is U-Net model.^10^ The U-Net model is a CNN architecture designed for semantic image segmentation, particularly in the medical imaging domain. It was introduced by Olaf Ronneberger, Philipp Fischer, and Thomas Brox in 2015 and has since become a widely used and influential model in the field of computer vision. The name “U-Net” is derived from the network's U-shaped architecture.^11^

Despite the success of the basic U-Net model, recent literature highlights critical limitations in existing segmentation approaches. For instance, excessive architectural depth can lead to overfitting, while shallow models may fail to capture complex anatomical features. Additionally, many studies overlook the trade-off between segmentation accuracy and computational cost, which is essential for deploying models in real-time clinical settings. The recent work by Meedeniya et al.^12^ emphasizes that model performance must be evaluated not only by accuracy but also by efficiency and generalizability across varied medical imaging conditions.

Furthermore, although various U-Net variants have been proposed, there remains a lack of systematic analysis comparing U-Net architectures of different depths and widths on the same medical dataset. This gap limits our understanding of how architectural configurations impact segmentation performance in practice. To address these challenges, this study proposes a comprehensive comparative evaluation of eight U-Net variants U-Net7, U-Net9, U-Net11, U-Net13 (depth-based), and U-Net16, U-Net32, U-Net64, and U-Net128 (width-based). These models are tested on a unified dataset of chest X-ray (CXR) images.

This study addresses three main research objectives. First, it investigates the impact of varying the number of layers in U-Net architectures on segmentation accuracy, aiming to determine whether increasing network depth leads to improved performance. Second, it examines how changes in the number of filters across layers (i.e., model width) affect both segmentation performance and training time. Lastly, the study seeks to identify which U-Net variant provides the most optimal balance between accuracy, computational efficiency, and applicability in real-world medical environments. To this end, eight different U-Net variants are implemented and evaluated on a chest radiography dataset using consistent training parameters. The models are compared using key performance metrics such as dice coefficient (DC), Intersection over Union (IoU), training time, and total parameters. The findings of this study aim to guide the selection of suitable U-Net configurations for medical image segmentation tasks, especially in settings that demand both high precision and practical deployability.

## Related works

Medical image analysis using deep learning has been the focus of extensive research in recent years. Among these, segmentation, classification, and hybrid methods combining both have played crucial roles in automated diagnosis. This section categorizes and analyzes the existing literature under three themes: (1) segmentation-only approaches, (2) classification-only methods, and (3) segmentation followed by classification.

### Segmentation-only approaches

U-Net and its variants have been widely adopted for medical image segmentation tasks. Alshbishiri et al.^13^ used a U-Net-based architecture to detect adenoid gland abnormalities in DICOM X-ray images, achieving a dice score of 0.74 for the adenoid region. Although effective, their method lacked broader architectural comparison. Woo et al.^14^ compared 2D and 3D U-Nets on brain MRI images and found 3D U-Net superior in accuracy but more resource-intensive, indicating limitations in scalability. Similarly, Yildiz et al.^15^ applied U-Net for multiclass segmentation in colon histology images but reported relatively low performance (dice: 57.08%), highlighting the challenge of segmenting complex tissues. Zheng et al.^16^ adapted U-Net for remote sensing image segmentation, achieving good generalization, though not focused on medical images. Ren et al.^17^ improved U-Net with residual modules for hippocampus segmentation, significantly enhancing accuracy (dice: 90.14%), yet their approach increases model complexity. Nimalsiri et al.^18^ developed the CXLSeg dataset with 243,324 frontal-view X-ray images and corresponding lung masks. They used a Spatial Attention U-Net (SA-UNet) for segmentation, achieving a dice score of 96.80% and IoU of 91.97%. Their study demonstrates that presegmentation improved CNN classification accuracy over unsegmented MIMIC-CXR images. However, they did not explore real-time efficiency or broader architectural comparisons. These works indicate the versatility of U-Net in segmentation tasks.

### Classification-only approaches

Several studies have used deep learning models solely for classification of medical conditions. For example, the study^19^ proposed a CNN-based framework to classify COVID-19 and pneumonia cases from CXR images. Although the classification performance was strong, this approach did not utilize segmentation, which may limit localization capabilities. Classification-only approaches tend to ignore fine-grained structural information, which can be crucial for interpretability and precision in diagnosis.

### Segmentation followed by classification

Combining segmentation with classification has been shown to improve diagnostic outcomes. For instance, this study^20^ proposed a system that first segments infected regions in X-ray images and then classifies the condition. This two-step approach provides both localization and diagnosis, increasing the clinical usefulness of the model. However, such hybrid systems may be computationally heavier and harder to optimize end-to-end.

Other studies employing image segmentation based on deep learning include the following studies. They are SegNet,^21^ Mask Regions with CNNs (R-CNN),^22^ DeepLab^23^ E-Net,^24^ LinkNet,^25^ Pyramid Scene Parsing Network (PSPNet),^26^ R-CNN,^27^ and FCNet.^28^

SegNet^4^ is a deep CNN architecture designed for pixel-wise semantic segmentation, particularly known for its encoder-decoder structure that helps in reducing computational complexity while retaining high segmentation accuracy. Mask R-CNN^22^ extends the Faster R-CNN model by adding a branch for predicting segmentation masks on each Region of Interest (RoI). It makes RoI highly effective, for instance, segmentation tasks. DeepLab^28^ employs dilated convolutions and a fully connected Conditional Random Field to achieve precise semantic segmentation by capturing multiscale contextual information. E-Net^29^ is an efficient neural network architecture optimized for real-time semantic segmentation on mobile and embedded devices. It includes balancing accuracy and computational load. LinkNet^30^ is a lightweight neural network architecture that uses residual connections to combine high-resolution feature maps from the encoder with the corresponding decoder layers. It facilitates fast and accurate semantic segmentation. Pyramid Scene Parsing Network^31^ introduces a pyramid pooling module that captures global context information from different subregions of an image. It includes enhancing the model's ability to perform accurate scene parsing. Regions with CNNs^27^ is one of the earliest models that successfully applied CNNs to object detection by proposing a method to extract regions of interest from images and classify them. FCN^28^ is a pioneering deep learning model for semantic segmentation, which replaces the fully connected layers in traditional CNNs with convolutional layers to produce dense predictions for each pixel.

The primary objective of this study is to evaluate the performance of image segmentation using new variants of the U-Net model, with the aim of enhancing diagnostic accuracy for medical practitioners. These variants include U-Net7, U-Net9, U-Net11, U-Net13, U-Net16, U-Net32, U-Net64, and U-Net128. The names of U-Net variants up to U-Net13 are based on their number of layers, while variants up to U-Net128 are distinguished by their number of filters. The quality of image segmentation is assessed using two key metrics: the DC and IoU. These metrics evaluate the accuracy of the overlap between the mask and the original image. The main contribution of this work is as follows.

Manual image segmentation, when conducted using computerized tomography (CT) images, can be time-consuming and lead to incorrect results for specialists. Therefore, there is a significant requirement to develop a segmentation method that can assist experts in disease diagnosis by using masks along with the original images. In this study, numerous models based on image segmentation have been developed. It includes enhancing the quality of segmentation from CT images. This study makes several notable contributions to the field of medical image segmentation, which can be summarized as follows:

1. This methodology has been applied specifically in the context of medical image segmentation. It demonstrates both novelty and potential impact within this field.
2. The proposed methodology has been rigorously evaluated against previously employed approaches. The results highlight its superior performance.
3. The quality and reliability of segmentation results have been significantly improved through refined segmentation models.
4. The study differentiates the U-Net variants based on the number of layers (for U-Net7 to U-Net13) and the number of filters (for U-Net16 to U-Net128). This approach allows for a systematic evaluation of how different architectural changes impact segmentation performance.
5. The proposed U-Net variants demonstrate significant potential in assisting radiologists and medical professionals. They enable timely and accurate diagnostic decisions through precise segmentation of medical images. This approach reduces manual effort and minimizes the potential errors associated with traditional methods.

## Methods

### Dataset acquisition

**Figure 1.**
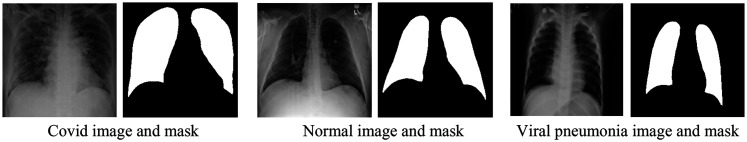
Image and corresponding mask samples included in the dataset.

In the pursuit of this research, the implementation of image segmentation has been carried out using CXR images sourced from a comprehensive dataset available on Kaggle. This study utilizes CXR images obtain from a publicly available dataset on Kaggle^32^ for patient detection. This dataset comprises CXR images corresponding to various conditions. It includes normal cases, viral pneumonia, and instances of COVID-19 positivity, each accompanied by their respective masks for segmentation. Specifically, the dataset includes 1200 images depicting COVID-19 positivity along with their corresponding masks, 1341 images portraying normal cases and their associated masks, and 1345 images representing viral pneumonia, each with its corresponding masks. The annotations associated with the CXR images were meticulously curated and thoroughly verified by experienced medical professionals. This diverse dataset allows for detailed exploration of image segmentation techniques across different medical conditions. It includes contributing to a more comprehensive understanding of the segmentation process in the context of CXR analysis. Each instance of an image in the dataset varies in dimensions. Examples of chest radiography images included in this dataset are illustrated in Figure 1.

### The process of workflow

**Figure 2.**
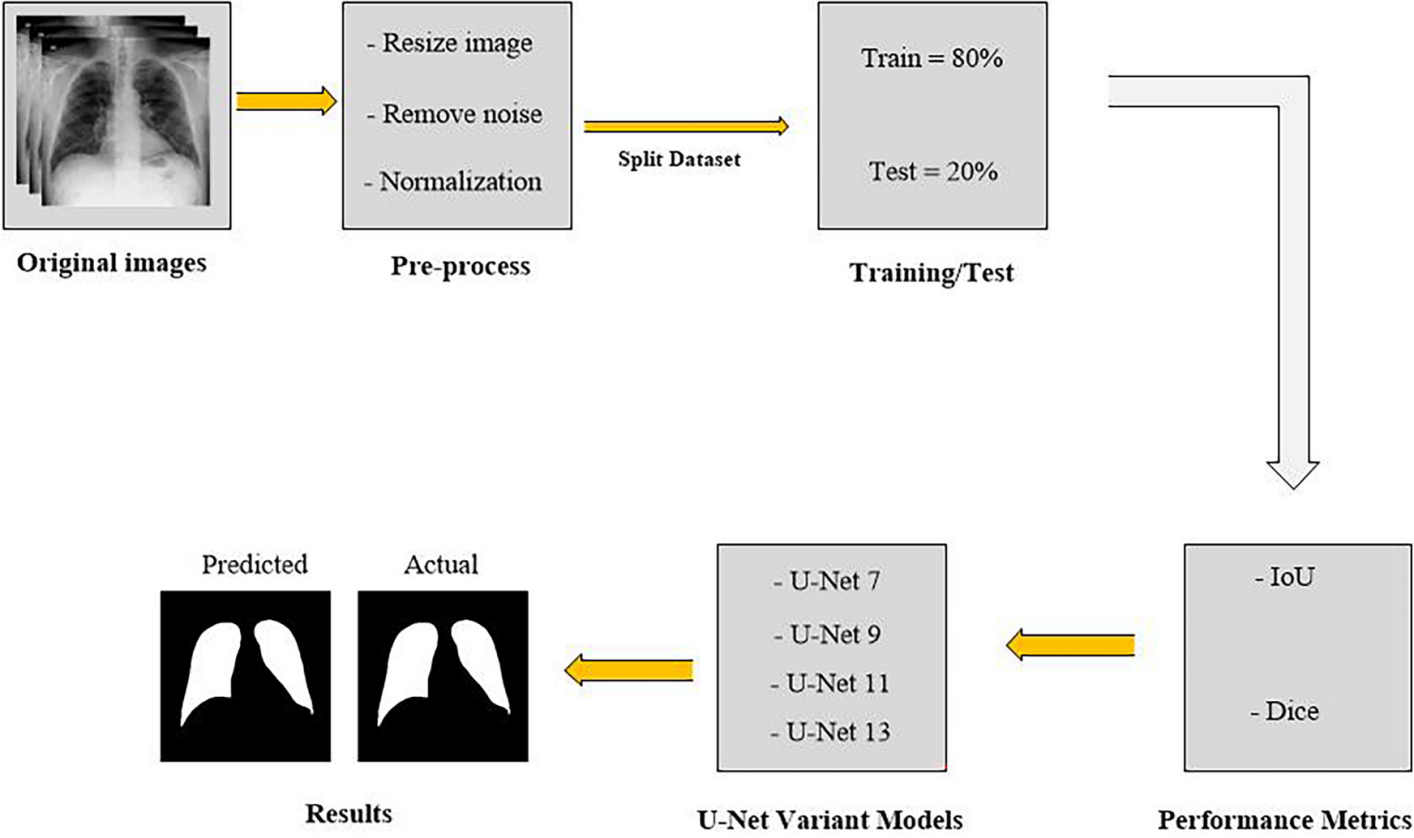
The flowchart of image segmentation via U-Net model.

The image processing workflow is illustrated in Figure 2. This flowchart illustrates the process of image segmentation using the U-Net9 model. It focuses on how images are pre-processed, split, and used in training, and how performance is evaluated. The process begins with a set of original medical images, which appear to be X-ray scans. These images undergo preprocessing, including resizing to a uniform dimension for consistency, noise removal to eliminate irrelevant data or artifacts, and normalization to adjust intensity values. For noise removal, a Gaussian filter with a kernel size of 3 × 3 and a standard deviation (σ) of 0.5 was applied. This method was selected due to its effectiveness in reducing Gaussian noise while maintaining edge integrity in medical images. Additionally, histogram normalization was used to improve feature visibility. These steps enhance image quality and ensure better performance during neural network training. After preprocessing, the dataset is split into training and testing sets. Unlike a conventional 80–20 split, this approach allocates 80% of the data for training and 20% for testing, prioritizing model evaluation. The segmentation task is performed using multiple U-Net variants, including U-Net 7, U-Net 9, U-Net 11, and U-Net 13. Each model is trained using 80% of the dataset. It includes learning to identify relevant patterns and features for accurate segmentation. The trained models are then tested on the remaining 20% of the dataset to evaluate their generalization capability. The performance of each model is assessed using key evaluation metrics. The IoU measures the overlap between the predicted and actual segmentation. It determines how accurately the model identifies the target regions. The DC quantifies the similarity between the predicted and actual segmentations. It includes focusing on precision and recall. The results are visualized using predicted and actual segmentation masks. By comparing these masks, the segmentation accuracy of different U-Net variants is analyzed, and it helps identify the most effective model for medical image segmentation.

### U-Net model

U-Net is a CNN architecture designed specifically for semantic segmentation tasks, particularly prevalent in medical image analysis. Developed by Olaf Ronneberger, Philipp Fischer, and Thomas Brox in 2015, the U-Net architecture has become widely influential.^33^ The model consists of a contracting path, which captures context and reduces spatial resolution through a series of convolutional and pooling layers. At the heart of U-Net lies a bottleneck that preserves high-resolution context information using skip connections. These connections link corresponding layers in the encoder and decoder, mitigating information loss during downsampling. The expanding path is responsible for upsampling feature maps. It employs transposed convolutions and concatenation operations to merge high-level semantic information with fine-grained spatial details. Notably, U-Net's distinctive feature is its use of skip connections, facilitating the recovery of spatial information during the upsampling process. The final layer often employs a convolutional layer with a softmax activation for multi-class segmentation. U-Net's ability to preserve high-resolution details and adapt to specific segmentation tasks makes it particularly effective for biomedical image segmentation, such as identifying organs, tumors, or abnormalities in radiological images. U-Net architecture is composed of a contracting path to capture context and a symmetric expanding path that enables precise localization. This contract-expand structure often referred to as an “encoder-decoder” U-shaped architecture. It offers inherent advantages in the field of medical image segmentation, particularly where training data are limited, and the semantic content is relatively uniform. The encoder stage functions as a typical contracting network, consisting of successive Block layers. To clearly describe the U-shaped structure, a Block is defined as performing a 3 × 3 convolution, followed by batch normalization and ReLU activation, effectively doubling the number of input features^34^:

(1) $$\mathrm{Block}(x)=\sigma (w_{bn}(w_{\mathrm{conv}}x+b_{\mathrm{conv}})+b_{bn})$$

(2) $$w_{bn}=\frac{\gamma }{\sqrt{\mathrm{Var}[x]}+\varepsilon }w$$

(3) $$b_{bn}=\frac{\gamma }{\sqrt{\mathrm{Var}[x]}+\varepsilon }(b-E[x])+\beta$$

In equations (1), (2), and (3), $w_{\mathrm{conv}}$ and $b_{\mathrm{conv}}$ represent the weight and bias of the 3 × 3 convolutional kernel, while $w_{bn}$ and $b_{bn}$ denote the weight and bias associated with batch normalization. The input features are represented by *x* and the activation function σ refers to ReLU. E[ *x* ] and Var[x] represent the mean and variance of the current batch, respectively, with γ and β being learnable parameters. Each stage of the network contains two Blocks, followed by a pooling layer. This sequential arrangement of layers effectively captures the necessary information about the target. However, during the training process, the neural network may experience issues such as slow convergence, gradient explosion, or difficulty in training. In such cases, batch normalization (BN) serves as an effective technique to address these challenges.

### Proposed U-Net9 model

**Figure 3.**
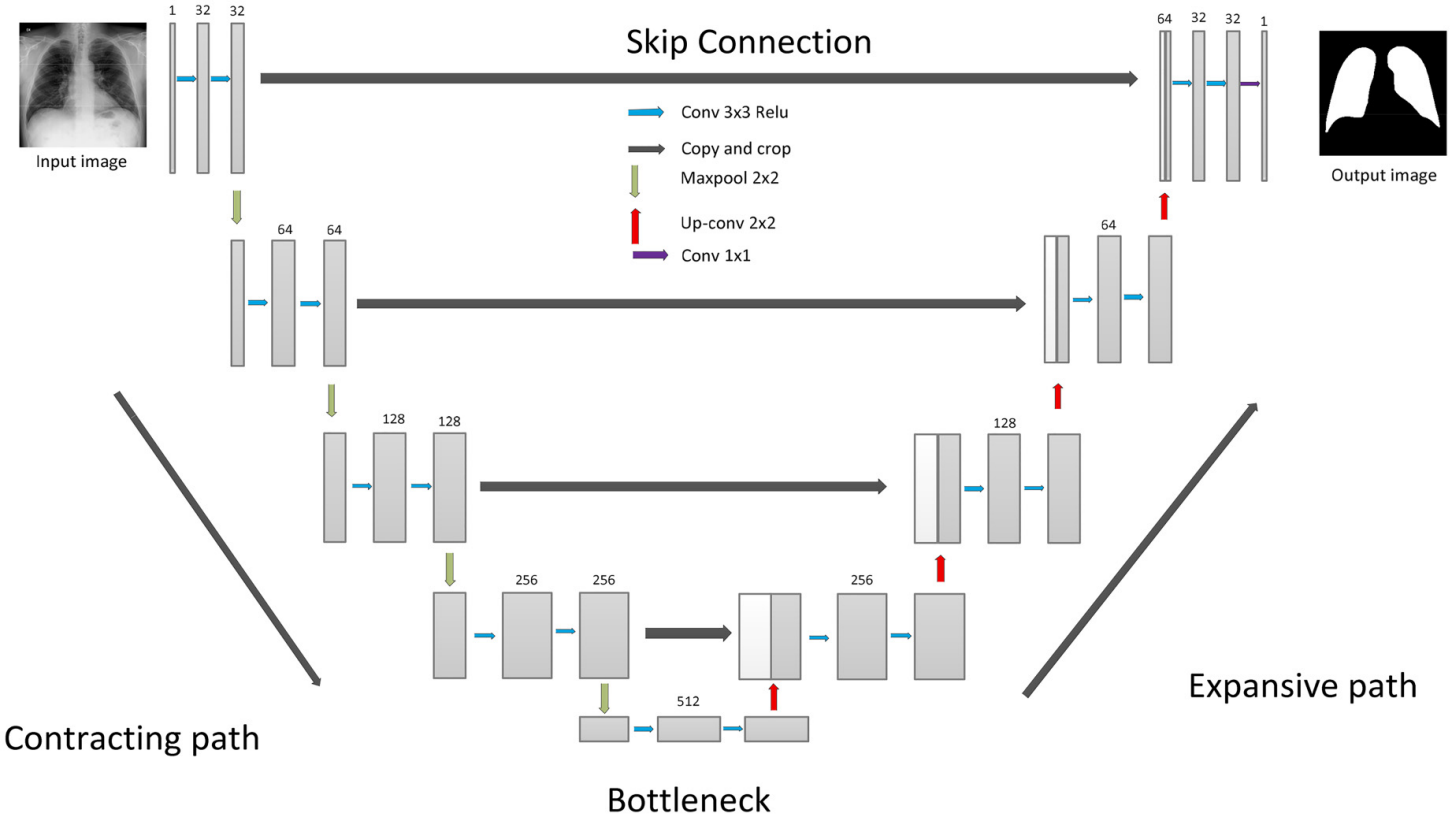
The proposed U-Net model.^35^

The proposed model, which is illustrated in Figure 3, named U-Net9, introduces a novel segmentation architecture characterized by nine layers. It draws inspiration from the foundational U-Net design. This model adheres to the classic U-Net structure, and it starts with a kernel size of 32. U-Net9 also incorporates a bottleneck at its core, strategically positioned to retain crucial high-resolution context information. This bottleneck helps preserve spatial details during processing. The decision to use nine layers, along with the selected kernel size, reflects a careful balance between computational efficiency and model expressiveness. Like the original U-Net, U-Net9 likely includes a combination of convolutional, max-pooling, and upsampling layers. However, it has a reduced overall layer count to decrease complexity. U-Net9 follows the U-Net's characteristic contracting path. In this path, the input image is progressively downsampled through convolutional layers and max-pooling operations. This captures context and features at multiple scales. The expanding path then upsamples these features back to the original resolution, with concatenations from corresponding layers in the contracting path. This refinement allows for precise localization of segmentation boundaries. This design makes the U-Net9 model effective for a variety of segmentation tasks. It also has the potential for adaptation to related tasks through fine-tuning or further modifications.

### Variants of U-Net model

**Figure 4.**
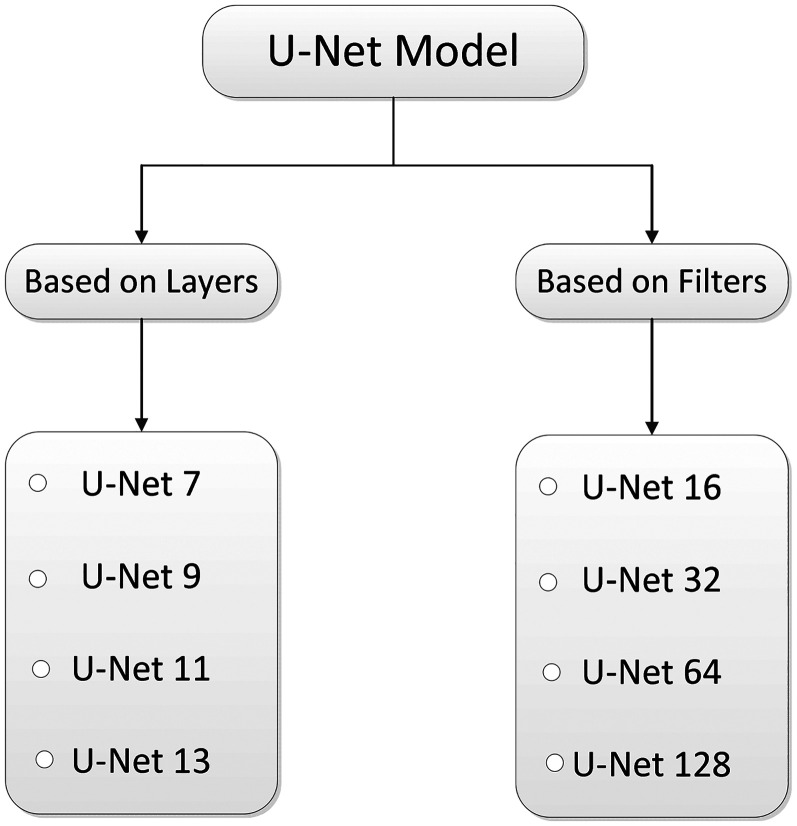
The variants of U-Net models.^36^

To systematically analyze the architectural impact on segmentation performance, U-Net model variants were categorized into two main groups in Figure 4. These categories are based on either the number of layers or the number of filters used in the model.

The first four models (U-Net7 to U-Net13) represent variants based on increasing depth (i.e., number of layers) while decreasing the initial number of filters. In contrast, the next four models (U-Net16 to U-Net128) keep the network depth constant (same as U-Net9) and vary the number of filters uniformly across all layers. This design helps in isolating the effects of depth versus filter width on the segmentation performance.

To provide a clearer understanding of the architectural configurations used in this study, it is summarized the main structural differences among the U-Net variants in terms of their number of convolutional layers and initial filter sizes.

Table 1 summarizes the architectural configuration of the U-Net variants evaluated in this study. The first group of models, from U-Net7 to U-Net13, differs primarily in the number of layers (i.e., network depth), while their initial number of filters decreases progressively from U-Net128 to U-Net16. This configuration allows us to analyze how increasing depth with fewer filters impacts segmentation performance and computational efficiency. In contrast, the second group, from U-Net16 to U-Net128, maintains the same number of layers as U-Net9 but varies in the number of filters used consistently across all layers. This enables the evaluation of network width and its influence on model complexity and accuracy. Such a design helps isolate the effects of depth and filter size, offering a clearer understanding of how architectural choices affect segmentation outcomes in medical imaging tasks.

**Table 1.** Number of layers and initial filter of U-Net variants.

Variants of U-Net model		
Model	Number of Layers	Initial Number of Filters
U-Net7	7	128
U-Net9	9	64
U-Net11	11	32
U-Net13	13	16
U-Net16	Fixed (same as U-Net9)	All layers 16
U-Net32	Fixed (same as U-Net9)	All layers 32
U-Net64	Fixed (same as U-Net9)	All layers 64
U-Net128	Fixed (same as U-Net9)	All layers 128

### Performance evaluation metrics for the U-Net model

To evaluate the algorithm's performance, the dataset was partitioned into training and test sets. The total dataset encompasses three distinct classes: COVID-19, normal, and viral pneumonia. The entire set of images was partitioned, allocating 80% for training purposes and reserving the remaining 20% for rigorous testing and evaluation. This meticulous splitting of images into distinct subsets ensures a robust evaluation of the algorithm's capability across diverse classes. It includes providing a comprehensive understanding of its effectiveness in handling COVID-19, normal, and viral pneumonia cases.

**Figure 5.**
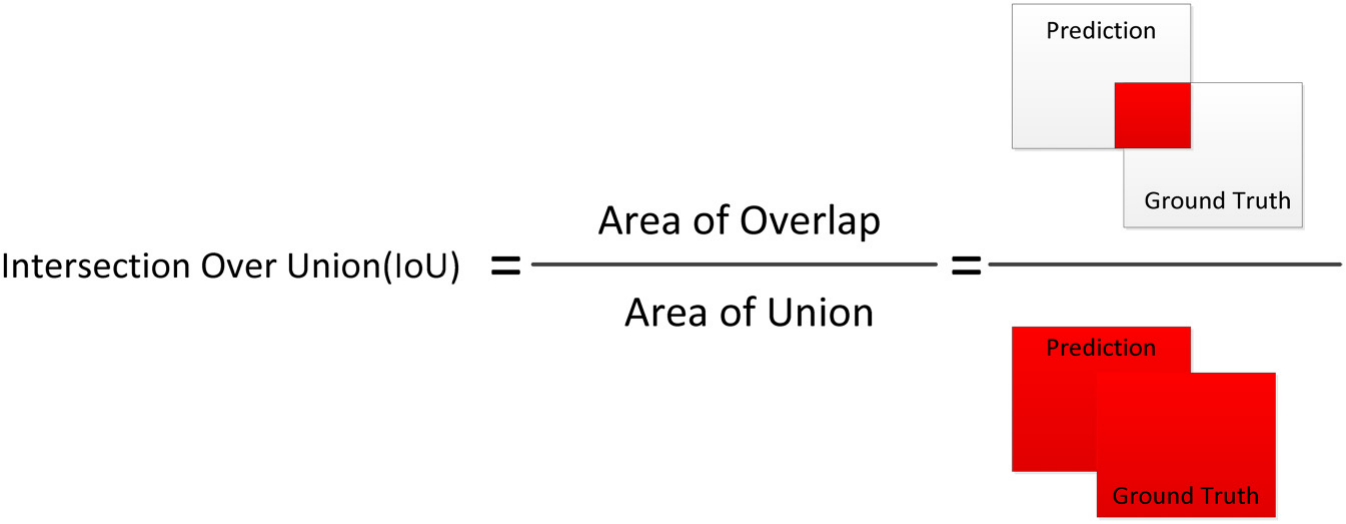
The graphical presentation for intersection over union.^13^

Figure 5 demonstrates the graphical presentation of the formula of IoU. To evaluate the efficacy of semantic segmentation, it was utilized as a quantitative quality assessment measure based on IoU and DC. These metrics are widely used in the literature for evaluating segmentation tasks due to their ability to quantify spatial overlap between predicted and ground truth masks.

More specifically, the DC emphasizes the similarity between the two sets by focusing on precision and recall, which is particularly useful in medical contexts where false negatives can be critical. On the other hand, IoU provides a stricter and more comprehensive measure of segmentation accuracy by accounting for both false positives and false negatives.

The IoU is calculated as the ratio of overlapping pixels between the actual and predicted labels (masks) to the total number of pixels in both labels. Mathematically, this measure is expressed as the intersection of the two masks divided by their union, providing a numerical representation of the degree of agreement between the predicted and actual segmentation. Mathematically, it can be represented in equation (4)^37^:

(4) $$\mathrm{IoU}=\frac{\mathrm{GroundTruth}\cap \mathrm{Prediction}}{\mathrm{GroundTruth}\cup \mathrm{Prediction}}=\frac{TP}{TP+FN+FP}$$

**Figure 6.**
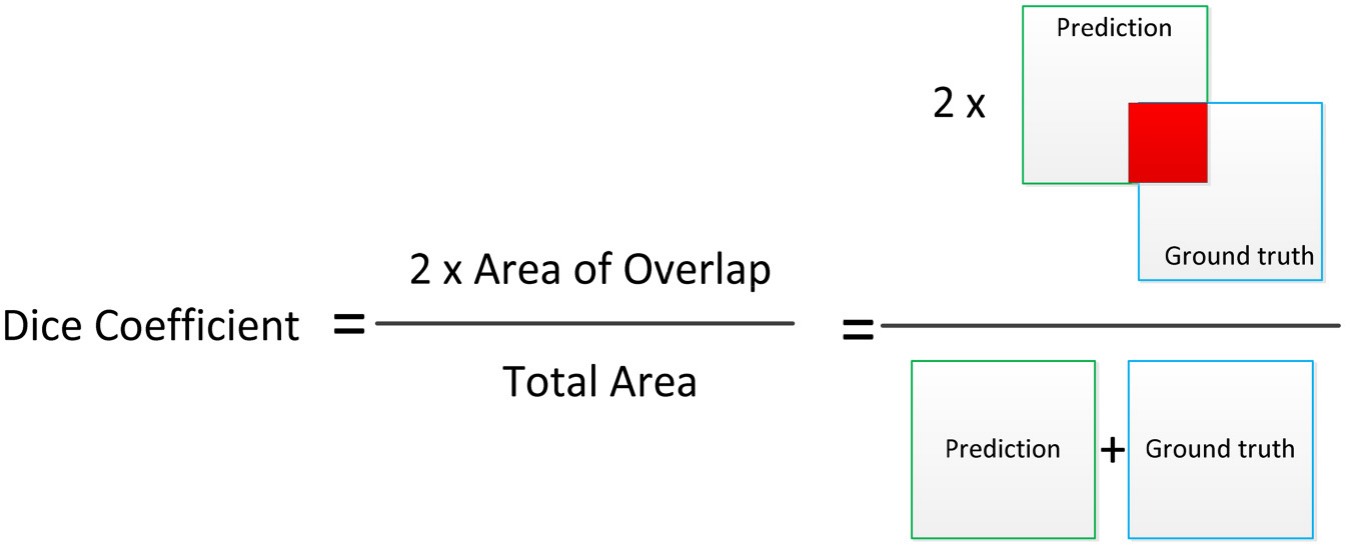
The graphical presentation for dice coefficient.^13^

Figure 6 illustrates the graphical presentation for DC. It is widely used metric for this purpose is the DC, which quantifies the overlap between the predicted and ground truth segmentations. In this context, true positive (TP) represents the number of pixels correctly classified as part of the target class, while false positive (FP) includes pixels incorrectly predicted as part of the target class, and false negative (FN) refers to pixels that belong to the target class but were not identified by the algorithm. Figure 6 provides helping to visualize the relationship between these values. This figure complements the mathematical definition presented in equation (5), where the DC is calculated^37^:

(5) $$\mathrm{Dice}=\frac{2\;\times \;TP}{\left(TP+FN)+(TP+FP\right)}$$

### Experimental setup for U-Net variants using CXR images

To ensure a fair and consistent evaluation across all models, each U-Net variant was trained under identical experimental settings. Table 2 summarizes the training parameters employed for all configurations.

**Table 2.** Experimental setup for U-Net variants using chest X-ray images.

	Training parameters of models					
Model	ImageSize	Epochs	BatchSize	Optimizer	LearningRate	Loss
U-Net7	256 × 256	10	20	Adam	0.0001	BinaryCrossentropy
U-Net9	256 × 256	10	20	Adam	0.0001	BinaryCrossentropy
U-Net11	256 × 256	10	20	Adam	0.0001	BinaryCrossentropy
U-Net13	256 × 256	10	20	Adam	0.0001	BinaryCrossentropy
U-Net16	256 × 256	10	20	Adam	0.0001	BinaryCrossentropy
U-Net32	256 × 256	10	20	Adam	0.0001	BinaryCrossentropy
U-Net64	256 × 256	10	20	Adam	0.0001	BinaryCrossentropy
U-Net128	256 × 256	10	20	Adam	0.0001	BinaryCrossentropy

All models were trained using CXR images resized to 256 × 256 pixels, which balances detail preservation and computational efficiency. A total of 10 epochs and a batch size of 20 were used during training to maintain consistency in learning dynamics and convergence behavior. The Adam optimizer was selected for its adaptive learning capabilities, with a learning rate fixed at 0.0001 to ensure stable gradient updates. For the loss function, binary cross-entropy was applied, which is well-suited for binary segmentation tasks such as distinguishing between anatomical regions and background. This standardized training setup allows for a direct comparison of performance across different U-Net depth levels, ranging from shallow (U-Net7) to deeper architectures (U-Net128), facilitating an analysis of how network complexity affects segmentation quality.

As shown in the table, all U-Net variants were trained under identical conditions: the same image size (256 × 256), optimizer (Adam), learning rate (0.0001), loss function (Binary Crossentropy), batch size (20), and number of epochs (10). This uniform setup was intentionally chosen to ensure a fair comparison between architectures. Since all models were trained equally, the superior performance of U-Net9 can be attributed to its architectural balance rather than training differences.

### Computational cost comparison of U-Net variants

In practical clinical applications, model accuracy alone is insufficient; computational efficiency is also a key factor. High-performing models that require excessive training time or computational resources may be impractical in real-time or resource-constrained medical environments. Therefore, we evaluated the computational cost of each U-Net variant in terms of the total number of trainable parameters and training time.

Table 3 presents a comparison of U-Net models from U-Net7 to U-Net128, highlighting how architectural changes affect computational requirements. All models were trained on the same dataset using identical hardware and training settings to ensure a fair comparison.

**Table 3.** Computational cost comparison of U-net variants based on parameter count and training time.

Total parameters and training time of models			
Model	ImageSize	Total params	Training time
U-Net7	256 × 256	7,696,193	118,80 min
U-Net9	256 × 256	7,778,017	120,11 min
U-Net11	256 × 256	7,781,761	55,50 min
U-Net13	256 × 256	7,945,457	27,15 min
U-Net16	256 × 256	52,993	19,98 min
U-Net32	256 × 256	210,945	28,58 min
U-Net64	256 × 256	841,729	64,40 min
U-Net128	256 × 256	3,362,817	172,25 min

The results in Table 2 indicate that model depth (U-Net7 to U-Net13) results in only a modest increase in parameters and training time, with U-Net9 slightly more demanding than U-Net7. Interestingly, U-Net11 and U-Net13 show reduced training times despite slightly higher parameter counts, possibly due to faster convergence or GPU memory handling efficiency. In contrast, U-Net16 to U-Net128 vary in the number of filters and thus show substantial differences in both total parameters and training time. U-Net128, although powerful, requires significantly more training time (172.25 min) and over 3 million parameters, making it less practical for time-sensitive applications. On the other hand, U-Net16 and U-Net32 offer a very low computational burden but at the cost of segmentation accuracy, as shown in “Results” section.

### Computational environment

All experiments and model training were conducted using the Google Colaboratory platform, which offers a cloud-based environment with GPU acceleration. Preliminary data preprocessing and organization steps were carried out on a local machine with the following specifications: Windows 10 operating system, Intel(R) Core(TM) i7–8550U CPU @ 1.80 GHz, and a 64-bit system architecture. This combination of local and cloud-based resources enabled efficient development, training, and evaluation of the proposed models.

## Results

Table 4 outlines the performance metrics for different U-Net models during both training and testing phases. Among the models, U-Net9 consistently emerges as the top performer. It includes achieving the highest scores for both DC (0.9645 in training and 0.9635 in testing) and IoU (0.9816 in training and 0.9808 in testing). Conversely, U-Net16 demonstrates the least favorable performance, recording the lowest DC (0.8859 in training and 0.8551 in testing) and IoU (0.9375 in training and 0.9197 in testing) across all models.

**Table 4.** Analysis of models results by using chest radiography dataset.

Model		Process		Dice		IoU
U-Net7		Train		0.9622		0.9804
		Test		0.9618		0.9795
**U-Net9**		Train		0.9645		0.9816
		**Test**		**0**.**9635**		**0**.**9808**
U-Net11		Train		0.9601		0.9793
		Test		0.9616		0.9800
U-Net13		Train		0.9504		0.9757
		Test		0.9536		0.9800
U-Net16		Train		0.8859		0.9375
		Test		0.8551		0.9197
U-Net32		Train		0.9204		0.9573
		Test		0.8824		0.9354
U-Net64		Train		0.9407		0.9686
		Test		0.9181		0.9557
U-Net128		Train		0.9565		0.9773
		Test		0.9329		0.9634

The findings indicate a pattern in which smaller U-Net architectures, specifically U-Net7 and U-Net9, demonstrate better performance when compared to their larger counterparts. Moreover, there is a noticeable decline as model complexity increases, evident in the decreasing scores from U-Net9 to U-Net128. It is crucial to consider the potential impact of overfitting, particularly noticeable in U-Net128, where there is a notable drop in performance between training and testing sets.

When selecting an optimal model, it is crucial to carefully consider the trade-off between model complexity and performance on new data. The U-Net9 model proves to be a well-balanced option. It includes exhibiting strong performance in both training and testing scenarios according to the provided metrics.

After the testing process, two types of images were obtained: actual and predicted. These images were compared to evaluate their similarity. A series of result images from different U-Net architectures, ranging from U-Net7 to U-Net128, were assessed using IoU and DC metrics. The comparison revealed that U-Net9 exhibited the best performance among the models, while U-Net16 demonstrated comparatively lower performance. The evaluation of various U-Net architectures, particularly the superior performance of U-Net9, indicates promising outcomes for this model. The findings highlight those architectures with fewer layers, such as U-Net9, not only yield better results but also offer benefits in terms of efficiency and reduced training times. Given these advantages, U-Net9 is well-suited for applications where time and computational efficiency are critical considerations.

**Figure 7.**
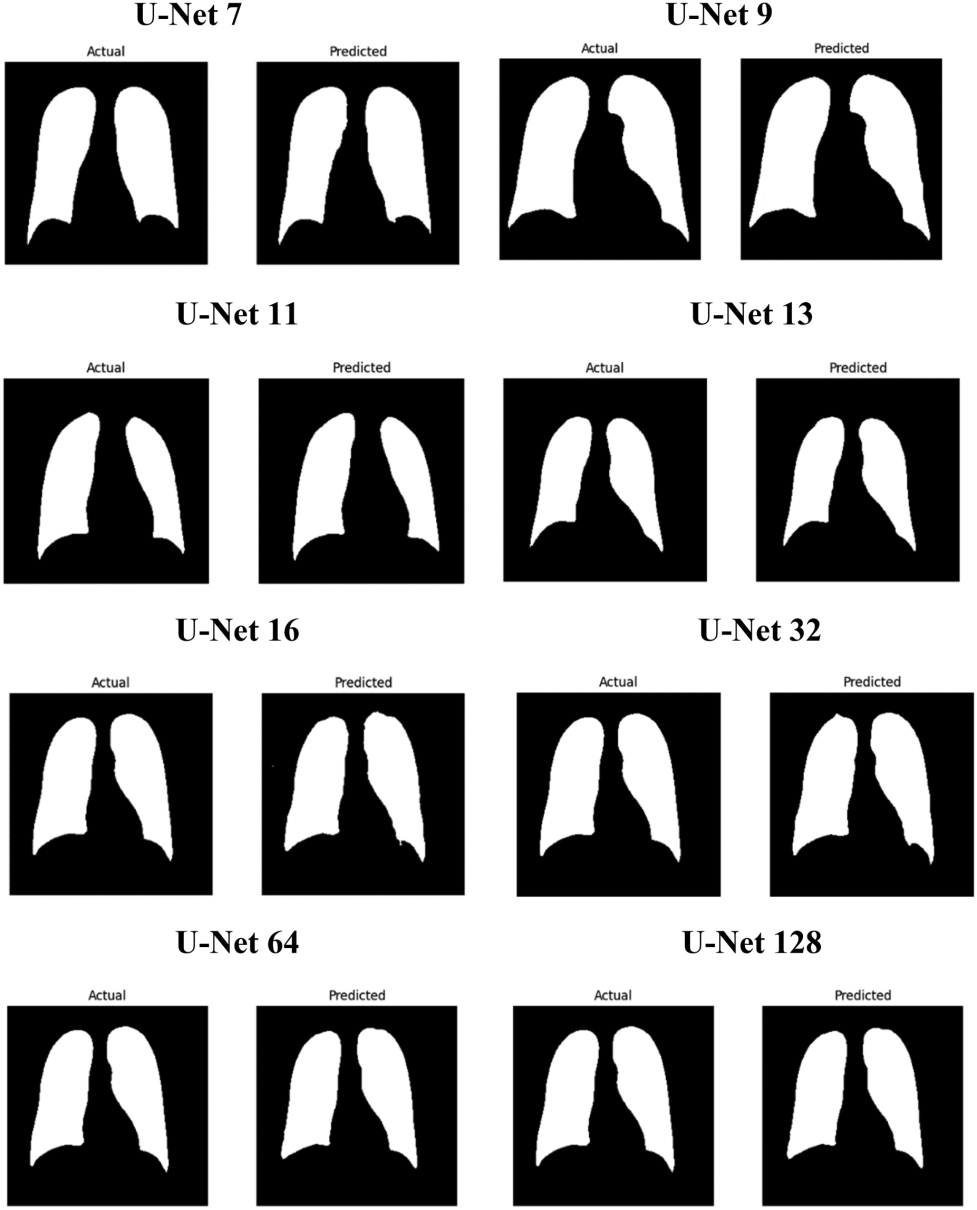
Illustrates the comparison of actual images along with their corresponding predicted images.

Figure 7 presents a comparison between the actual (ground truth) and predicted segmentation results for various U-Net model variants, including U-Net7, U-Net9, U-Net11, U-Net13, U-Net16, U-Net32, U-Net64, and U-Net128. Each row in the figure shows the actual segmentation mask (left) and the predicted segmentation mask (right) generated by the corresponding U-Net model. U-Net9 emerges as the most effective model among the variants. It provides the closest match to the actual mask, with high accuracy, sharp boundaries, and minimal errors. This is consistent with the performance metrics, where U-Net9 achieved the highest scores. The models with lower U-Net numbers (like U-Net7) generally offer decent segmentation but lack the precision seen in models with higher numbers. As the number increases (especially in U-Net9, U-Net11), the models generally improve in capturing fine details and in maintaining edge accuracy. As the model depth increases to U-Net16 and U-Net32, the segmentation quality remains high, but slight variations begin to emerge. These variations are more pronounced in U-Net64 and U-Net128, where a diminishing return in segmentation quality is observed. These deeper models, while still capable of performing the task, tend to produce less detailed segmentations, potentially due to overfitting or the complexity of managing a higher number of layers. This suggests that an optimal balance between model depth and performance exists, where models like U-Net9 or U-Net16 offer a more efficient and accurate solution for image segmentation tasks. This analysis supports the conclusion that while deeper models can be effective, there is optimal depth for U-Net architecture, with U-Net9 providing the best balance of accuracy and detail for the segmentation tasks presented here.

Table 5 presents a comparison of image segmentation models, focusing on variants of the U-Net architecture. Two key metrics are used: the DC and the IoU. These measures assess how accurately a model segments images by comparing predicted and actual masks. The proposed U-Net9 model achieves a DC of 0.96 and an IoU of 0.98. These values are the highest reported in the table. In contrast, Hu et al. applied a standard U-Net and obtained a DC of 0.60, with no IoU provided. This indicates modest performance. Harsh et al. utilized Attention U-Net, recording an IoU of 0.94, though no DC was included. Tatli et al. evaluated U-Net11, achieving a DC of 0.95 and an IoU of 0.91. These results are strong but fall slightly below U-Net9. Conversely, Yıldız et al. reported the lowest scores, with a DC of 0.57 and an IoU of 0.48. Such findings demonstrate the superior capability of U-Net9 among the listed studies.

**Table 5.** Comparison of relevant studies for automatic image segmentation from images and masks using U-Net model.

Reference		Used method		Dice		IoU
Hu et al.^22^		U-Net		0.60		_
Harsh et al.^23^		Attention U-Net		0.94		_
Dedeoglu et al.^38^		U-Net (FDL)		0.90		0.86
Tatli et al.^36^		U-Net11		0.95		0.91
Zadeh et al.^39^		U-Net++		0.90		_
Wang et al.^40^		DC U-Net		0.94		_
Yin et al.^2^		DFBU-Net		**0**.**96**		_
Ren et al.^17^		U-Net		0.90		_
Hong et al.^1^		M2ANet		0.75		0.81
Yıldız et al.^15^		U-Net		0.57		0.48
Liu et al.^41^		ResAttU-Net		0.83		0.93
Nimalsiri et al.^18^		SA-UNet		0.96		0.91
**Proposed study**		**U-Net9**		**0**.**96**		**0.98**

Additional models in the table reveal a range of performances, yet none surpass U-Net9 overall. Yin et al. tested DFBU-Net, which matched U-Net9's IoU of 0.96; however, DC was not reported. This limits a complete comparison. Dedeoglu et al. employed U-Net (FDL), yielding a DC of 0.90 and an IoU of 0.86. These figures reflect reliable but lesser performance. Liu et al. assessed ResAttU-Net, with a DC of 0.83 and an IoU of 0.93, indicating moderate success. Hong et al. used M2ANet, scoring a DC of 0.75 and an IoU of 0.81, which are notably lower. Zadeh et al. applied U-Net++, achieving a DC of 0.90, though IoU lack complete data. Wang et al. tested DC U-Net, reporting an IoU of 0.94 without a DC. Despite these competitive results, U-Net9 consistently excels in both metrics. This evidence underscores its effectiveness as a leading model for image segmentation tasks. Nimalsiri et al. proposed SA-UNet, which achieved a high dice score of 0.96 and an IoU of 0.9. This demonstrates strong segmentation performance. These results suggest that the model excels in both dice and IoU, making it highly effective for medical image segmentation tasks.

### Limitations and gaps of work

While the study provides valuable insights into the segmentation performance of U-Net variants and highlights the superiority of the U-Net9 model, several important limitations should be acknowledged to contextualize the findings. Although the results are promising, they are derived from experiments conducted under controlled conditions, and their applicability to broader clinical or real-world scenarios has not yet been fully explored.

- First, the experiments were conducted on a single publicly available CXR dataset (Kaggle), which may restrict the generalizability of the findings to other medical imaging modalities such as CT or MRI, or even other X-ray datasets with different characteristics.
- Second, the research exclusively focused on U-Net variants, without exploring other advanced segmentation architectures such as attention-based U-Nets, U-Net++, DeepLab, or hybrid models that might offer complementary or superior performance under different conditions.
- Third, no statistical significance tests (e.g., *p*-values or confidence intervals) were performed to formally validate the observed differences in performance among the compared models, which limits the strength of comparative conclusions.

Addressing these limitations in future work will be essential to enhance the robustness, generalizability, and clinical readiness of the proposed models. Broader validation across diverse datasets and imaging modalities, as well as comparative analysis with other state-of-the-art architectures, will help solidify the practical contribution of this research.

## Discussion

This comprehensive study evaluated the performance of eight distinct U-Net models. The evaluation utilized a carefully prepared CXR dataset. Key performance metrics were employed to assess these models. The results demonstrated that the U-Net9 model performed exceptionally well. Specifically, it achieved a DC of 0.98 and an IoU of 0.96 on the dataset. These values positioned U-Net9 as the most effective model among those tested. The naming of the models reflects distinct architectural features. U-Net7, U-Net9, U-Net11, and U-Net13 are differentiated by their layer configurations. This design allowed us to evaluate how different depths (U-Net7 to U-Net13) influence segmentation accuracy, revealing that moderate depth (U-Net9) achieved the best results. In contrast, U-Net16, U-Net32, U-Net64, and U-Net128 are defined by the number of filters in their structures. These variants helped assess how increasing filter width affects both model performance and training time. Results indicated diminishing returns beyond U-Net9, with larger models incurring higher computational costs without substantial accuracy gains. The study highlighted the importance of the DC and IoU as essential metrics. These measures evaluate the quality of overlap between the predicted mask and the original image. Consequently, they play a critical role in determining the accuracy of segmentation outcomes. The U-Net9 model exhibited robust performance in segmenting CXR images. This suggests its suitability for biomedical applications involving CXRs. Furthermore, when compared to existing methods in the literature, U-Net9 demonstrated superior capabilities. Based on these findings, the study recommends U-Net9 as a strong alternative to the traditional U-Net model. The U-Net9 model emerges as an optimal choice for image segmentation tasks. It offers clear advantages over the other models evaluated in this study. Among all models tested, U-Net9 offered the most favorable trade-off between accuracy, computational efficiency, and clinical applicability, fulfilling the core goal of identifying a practical solution for real-world use.

The U-Net9 model, which showed the best segmentation performance, can support radiologists by clearly highlighting regions of interest in CXR images. This can help detect abnormalities more quickly and accurately. That is reducing the risk of human error. In clinical practice, using U-Net9 as a preprocessing tool could speed up diagnosis and assist in treatment planning by providing precise lung or disease area boundaries. Its efficient performance and low complexity also make it suitable for use in real time. In summary, the U-Net9 model stands out as an optimal choice for image segmentation tasks, offering superior performance compared to the other models considered. In future studies, it is planned to integrate multimodal medical images such as combining CXRs with CT or MRI scans to improve segmentation performance. It is also suggested testing advanced U-Net architectures that include attention mechanisms or residual connections to further enhance accuracy. Additionally, evaluating these models on larger, more diverse, and real-world clinical datasets will help test their generalizability.

## Conclusion

This study presented a comparative analysis of eight U-Net-based deep learning architectures for the segmentation of CXR images. The objective was to identify a model that offers both high segmentation performance and computational efficiency. Among all tested architectures, the U-Net9 model achieved the most successful results. It recorded a DC of 0.9645 during training and 0.9635 during testing. Similarly, it reached an IoU score of 0.9816 in training and 0.9808 in testing. The findings indicate that increasing the number of layers or filters does not always lead to performance improvements. On the contrary, excessively complex architectures may cause overfitting and lead to higher computational costs. The U-Net9 model provided the optimal balance between accuracy and model complexity. Compared to existing methods in the literature, U-Net9 demonstrated superior or highly competitive performance. This makes it a promising tool for real-time clinical applications, particularly in settings with limited computational resources. Its ability to accurately segment medical images can assist radiologists by enhancing diagnostic speed and reliability. In future studies, the model will be tested on multimodal datasets, including CT and MRI scans. Additionally, incorporating attention mechanisms or hybrid model designs will be considered to further improve segmentation performance.
